# Ecotone-Driven Vegetation Transitions Reshape Soil Nitrogen Cycling Functional Genes in Black Soils of Northeast China

**DOI:** 10.3390/biology14111474

**Published:** 2025-10-23

**Authors:** Junnan Ding, Yingjian Wang, Shaopeng Yu

**Affiliations:** 1 Heilongjiang Province Key Laboratory of Cold Region Wetland Ecology and Environment Research, Harbin University, Harbin 150086, China; ding.junnan@163.com; 2 School of Chemistry and Molecular Bioscience, Queensland University, Brisbane, QLD 4072, Australia; wyingjian12@gmail.com

**Keywords:** forest–wetland ecotone, black soils, nitrogen cycling, functional genes, microbial community assembly

## Abstract

**Simple Summary:**

Understanding how vegetation transitions influence soil microbial processes is essential for predicting nutrient cycling and greenhouse gas dynamics in ecotone ecosystems. In this study, we examined soils along a forest–wetland gradient in Northeast China, where fertile black soils serve as both agricultural and ecological resources. By integrating analyses of nitrogen-cycling functional genes, microbial diversity, community assembly, ecological networks, and predicted metabolic functions, we revealed that vegetation transitions restructure microbial communities through hydrological and biogeochemical heterogeneity rather than a simple linear gradient. Forest soils exhibited greater microbial diversity, more complex network connectivity, and higher potentials for nitrogen fixation and nitrification under oxic conditions. In contrast, wetland and edge soils harbored denitrification-enriched taxa and stronger carbon–nitrogen coupling under fluctuating redox states, indicating enhanced capacity for N_2_O reduction and metabolic resilience. The results also demonstrate that the wetland edge acts as a functional hotspot where aerobic and anaerobic processes coexist, and that hydrological and nutrient variability jointly shape microbial assembly, interaction networks, and functional stability. Overall, this study provides mechanistic insights into how vegetation-driven transitions regulate nutrient turnover and greenhouse gas fluxes, offering a scientific basis for the sustainable management of black-soil ecotones under changing environmental conditions.

**Abstract:**

Forest–wetland ecotones are transitional ecosystems characterized by pronounced hydrological and biogeochemical heterogeneity, yet the microbial mechanisms regulating nutrient cycling in these zones remain insufficiently understood. This study investigated how vegetation transitions across a forest–wetland ecotone in the black-soil region of Northeast China shape soil microbial communities and nitrogen–cycling functions. Soils were collected from four vegetation types: mixed forest (MF), coniferous forest (CF), wetland edge (WE), and natural wetland (NW). Quantitative PCR was used to quantify key nitrogen–cycling functional genes (*nifH*, *amoA*, *amoB*, *norB*, *nosZ*), and PICRUSt2 was applied to predict microbial functional potentials. Forest soils (MF and CF) exhibited higher microbial diversity, stronger network connectivity, and greater abundances of *nifH* and *amoA*, indicating enhanced nitrogen fixation and nitrification under oxic conditions. In contrast, wetland soils harbored denitrification-enriched communities with higher *norB* and *nosZ* abundances but lower diversity. The WE vegetation type acted as a functional hotspot where alternating oxic–anoxic conditions facilitated the coexistence of nitrifiers and denitrifiers, thereby enhancing carbon–nitrogen coupling and functional resilience. Redundancy and Mantel analyses identified soil organic carbon, total nitrogen, water content, and enzyme activities as major environmental drivers of microbial structural and functional variation. This study reveals that vegetation transitions reorganize microbial community assembly and nitrogen-cycling functions through hydrological and biogeochemical heterogeneity, providing mechanistic insights into nutrient turnover and ecological regulation in black-soil ecotones.

## 1. Introduction

Forest–wetland ecotones are transitional ecosystems characterized by pronounced environmental heterogeneity resulting from periodic fluctuations in hydrology, redox potential, and organic matter accumulation [1]. Such complex environmental gradients create diverse microhabitats that sustain high biodiversity and provide essential ecosystem services, including carbon storage, nutrient retention, water regulation, and climate buffering [2]. However, ecotones often exhibit limited resilience to external disturbances. Their strong edge effects and high sensitivity to climate change, land-use conversion, and hydrological alteration make them vulnerable hotspots of ecological instability [3]. For instance, in Northeast China, long-term hydrological changes such as groundwater table rise and artificial drainage have disrupted the balance between forests and wetlands, leading to shifts in vegetation structure and ecosystem functionality [4]. These large-scale transformations highlight the need to better understand the biogeochemical processes that underpin ecosystem dynamics in ecotones.

Because soil biogeochemical processes are tightly coupled with hydrological and redox conditions, forest–wetland ecotones are expected to exhibit strong ecological feedback under future environmental change [5]. Previous studies have shown that variations in soil moisture and nutrient availability play decisive roles in regulating microbially mediated nutrient cycling and greenhouse gas fluxes [6]. Thus, investigations of ecotone ecosystems should not only focus on vegetation dynamics but also on the microbial communities that drive soil processes and ecosystem functioning [7]. Soil microorganisms are central players in the nitrogen (N) cycle, influencing soil fertility, plant productivity, and greenhouse gas emissions [8]. The major microbial processes involved in the N cycle nitrogen fixation, nitrification, and denitrification—are commonly tracked using functional marker genes such as *nifH*, *amoA*, *norB*, and *nosZ* [9,10]. In recent years, additional pathways such as anaerobic ammonium oxidation (anammox), complete ammonia oxidation (comammox), and the functional distinction between *nosZ* clades I and II have been recognized as crucial components of the N cycle, substantially expanding its scope and affecting the capacity of soils to act as sinks or sources of nitrous oxide (N_2_O) [11,12]. Among them, anammox plays a particularly important role in wetland and peat-influenced systems, where low-redox conditions favor alternative anaerobic nitrogen pathways [13,14]. In this study, we focused on the widely applied functional genes *nifH*, *amoA*, *norB*, and *nosZ* as representative indicators of key nitrogen-cycling processes, while acknowledging that other pathways (e.g., anammox, comammox, *nosZ* clade II) remain critical directions for future research [15]. It is important to note that gene abundance represents the potential for specific processes but does not directly equate to process rates or actual N_2_O fluxes.

Microbial diversity and community composition also play vital roles in maintaining ecosystem functions [16]. Forest soils typically harbor aerobic decomposers and nitrifiers, while wetland soils are dominated by anaerobic taxa such as denitrifiers and methanogens [17]. Consequently, vegetation transitions along forest–wetland ecotones are expected to reorganize microbial assemblages, alter nitrogen-cycling pathways, and influence N_2_O emissions [18]. For example, periodic wetting and drying cycles in ecotones can lead to transient N_2_O emission peaks, whereas persistently saturated wetlands tend to favor complete denitrification to N_2_, thereby reducing net emissions [19]. Beyond community diversity, microbial assembly mechanisms and co-occurrence network structures determine how microbial communities respond to environmental transitions [15]. Deterministic environmental factors such as moisture, redox status, and nutrient gradients interact with stochastic processes such as dispersal limitation and ecological drift to shape microbial communities, while vegetation transitions further restructure microbial co-occurrence networks by altering connectivity, modularity, and keystone taxa [20,21]. Moreover, predictive functional profiling provides insight into how microbial metabolism adapts to changing carbon, nutrient, and redox conditions, offering a mechanistic understanding of ecosystem functioning under transitional environments [22,23]. Previous studies have examined vegetation–soil interactions and microbial processes in forest–wetland ecotones [3,17,24], but few have focused on the globally significant black soils of Northeast China. Unlike other ecotones, these Mollisols are characterized by exceptionally high organic matter and nitrogen contents, making them both agriculturally productive and highly sensitive to hydrological alteration. This unique background provides an ideal context to explore how vegetation transitions reorganize microbial communities and nitrogen cycling functions.

The objectives of this study were to (i) quantify changes in the abundance of nitrogen-cycling functional genes across vegetation types, (ii) assess microbial diversity, community composition, assembly processes, and ecological networks along the ecotone, and (iii) identify key soil properties and enzyme activities that drive microbial structural and functional variations. We hypothesized that vegetation transitions across the forest–wetland ecotone would induce a systematic reorganization of microbial communities and nitrogen-cycling capacities, primarily driven by variations in hydrological and nutrient regimes. By integrating molecular quantification, enzyme activity measurements, and multivariate statistical analyses, this study advances the understanding of how vegetation-mediated environmental heterogeneity regulates microbial assembly and nitrogen transformation processes. These findings provide a mechanistic foundation for improving nutrient management, mitigating greenhouse gas emissions, and guiding the ecological restoration of black-soil ecosystems.

## 2. Materials and Methods

### 2.1. Study Area and Sampling Design

The study was conducted in the Wuyiling Forestry Bureau of the Lesser Khingan Mountains, Heilongjiang Province, Northeast China (48°33′–48°50′ N, 129°00′–129°30′ E), a region characterized by black soils (Mollisols) with high organic matter and strong sensitivity to hydrological changes [25]. The area experiences a temperate continental monsoon climate, with a mean annual temperature of −1.1 °C, a frost-free period of approximately 97 days, and mean annual precipitation of about 584 mm, concentrated in summer [26]. Along a continuous ecological transition from upland forests to permanently waterlogged wetlands, we selected four representative vegetation types as points along this natural transition rather than as independent treatments. These included (Figure S1): mixed forest (MF), dominated by *Betula platyphylla*, *Larix olgensis*, and *Picea jezoensis*; coniferous forest (CF), primarily composed of *Larix olgensis* with understory shrubs such as *Lonicera caerulea* and *Spiraea salicifolia*; wetland edge (WE), characterized by transitional species including *Alnus sibirica*, *Syringa reticulata*, and *Prunus padus*; and natural wetland (NW), dominated by hygrophilous plants such as *Carex schmidtii*, *Deyeuxia angustifolia*, *Sanguisorba tenuifolia*, and *Filipendula palmata*. These vegetation types co-vary with differences in topography (upland to depression), hydrological regime (well-drained to waterlogged), soil development (mature forest soils to hydric soils), and redox conditions (oxic to anoxic), thereby collectively representing the forest–wetland ecotone and providing an ideal setting to examine microbial structural and functional transitions.

### 2.2. Soil Sampling

Soil sampling was conducted from 15 to 20 July 2022, in accordance with the Chinese national standard for soil sampling (GB/T 36199-2018) [27]. For each vegetation type, six 40 m^2^ square plots (5 × 8 m) were randomly established at least 50 m apart along the forest–wetland transitions to capture within-type heterogeneity and reduce potential spatial autocorrelation. Randomization was performed using GPS-based stratified random sampling. Within each plot, ten soil cores (0–20 cm) were collected along an S-shaped transect, homogenized, and pooled into one composite sample. This approach is widely applied in microbial ecology to minimize small-scale spatial heterogeneity and obtain representative samples. Sterile PVC augers were used and disinfected with 75% ethanol between plots to prevent cross-contamination. Each composite sample was treated as one independent plots, yielding a total of 24 independent samples (4 vegetation types × 6 replicates). In the laboratory, soils were sieved through a 2 mm mesh to remove coarse debris and plant residues. One portion of fresh field-moist soil was stored at 4 °C and used for enzyme activity assays within 72 h of collection, while the other portion was air-dried for physicochemical analyses. A third portion was stored at −80 °C for DNA extraction.

### 2.3. Soil Physicochemical Properties and Enzyme Activities

Soil pH was measured in a 1:2.5 soil–water suspension using a calibrated pH meter [28]. Soil water content (SWC) was determined gravimetrically after drying at 105 °C for 24 h. Soil organic carbon (SOC) and total nitrogen (TN) were analyzed with an Elementar Vario EL elemental analyzer [29,30]. NH_4_^+^-N and NO_3_^−^-N were extracted with 2 M KCl (soil:solution = 1:5, *w*/*v*) by shaking for 1 h at 25 °C and determined with a continuous flow analyzer (Seal AA3, Norderstedt, Germany) [30,31]. Available phosphorus (AP) was extracted with 0.5 M NaHCO_3_ using the Olsen-P method, and quantified colorimetrically, while available potassium (AK) was measured with 1 M ammonium acetate (NH_4_OAc) extraction and flame photometry [32,33]. Enzyme activities were measured under controlled incubation conditions. Urease activity was determined by incubating 5 g fresh soil with 10 mL 10% urea solution at 37 °C for 2 h, followed by colorimetric quantification of NH_4_^+^ released [34]. β-glucosidase activity was determined by incubating 2 g fresh soil with 5 mL 25 mM p-nitrophenyl-β-D-glucopyranoside at 37 °C for 1 h and measuring the released p-nitrophenol at 410 nm [35]. Cellulase activity was determined by incubating 2 g soil with 10 mL 1% carboxymethyl cellulose (CMC) solution at 37 °C for 24 h, followed by quantification of reducing sugars with the DNS method at 540 nm [36]. All assays were performed in triplicate with appropriate blanks. The detailed values are presented in Supplementary Table S1.

### 2.4. DNA Extraction and Quantification of Functional Genes

Soil DNA was extracted from 0.5 g fresh soil using the E.Z.N.A.^®^ Soil DNA Kit (Omega Bio-Tek, Norcross, GA, USA). DNA integrity was verified by 1% agarose gel electrophoresis, and concentration and purity were measured using NanoDrop (Thermo Fisher Scientific, Waltham, MA, USA) with A260/A280 ratios recorded to confirm purity. Quantitative PCR (qPCR) assays targeted nitrogen cycle genes: *nifH* (nitrogen fixation), bacterial and archaeal *amoA*, *amoB* (nitrification), *norB* (denitrification intermediate), and *nosZ* (N_2_O reduction) [37,38,39]. Amplification conditions were as follows: 95 °C for 3 min; 40 cycles of 95 °C for 15 s, 55–60 °C (gene-specific annealing temperatures) for 30 s, 72 °C for 30 s. Specificity of amplification was confirmed by melting curve analysis and verification of expected amplicon sizes through agarose gel electrophoresis, following established protocols. Reaction mixtures (20 μL) contained 10 μL SYBR Green Master Mix, 0.4 μL of each primer (10 μM), 2 μL DNA template, and nuclease-free water. All reactions were performed in triplicate on a Bio-Rad CFX96 system (Bio-Rad, Hercules, CA, USA). Standard curves constructed from plasmid DNA achieved R^2^ > 0.98 and amplification efficiencies between 90–105%. It should be noted that all functional gene abundances reported in this study were experimentally quantified by qPCR, whereas PICRUSt2 predictions were used solely for inferring metabolic pathway profiles and not for estimating gene copy numbers.

### 2.5. Microbial Community Profiling

The bacterial 16S rRNA V3–V4 region was amplified with primers 341F/806R and sequenced using the Illumina MiSeq platform (2 × 300 bp). Raw sequences were processed with QIIME2 and DADA2 to generate amplicon sequence variants (ASVs). Taxonomy was assigned against SILVA 138. Alpha diversity indices (Shannon, Chao1) and beta diversity metrics (Bray–Curtis dissimilarity) were calculated based on the ASV table. All indices were computed using the “vegan” package in R v4.3.2 [40]. To identify significantly different taxa among vegetation types, Linear discriminant analysis Effect Size (LEfSe) was applied with an LDA score threshold of 2.0, following the method of Segata et al. [41]. All raw sequencing data have been deposited in the NCBI Sequence Read Archive (SRA) under BioProject accession number PRJNA1271745. The corresponding OTU/ASV table is provided in the Supplementary Materials (Supplementary Table S2).

### 2.6. Community Assembly and Network Analyses

Microbial community assembly processes were inferred using a null model framework that combined the β-nearest taxon index (βNTI) and the Bray–Curtis-based Raup–Crick metric (RC_bray_) to distinguish deterministic and stochastic drivers of community composition [42]. Co-occurrence networks were constructed separately for each vegetation type based on Spearman correlations among ASVs. Only statistically significant correlations were retained to represent potential ecological associations. Network robustness was evaluated using natural connectivity (*η*), which reflects the overall structural stability of the network under progressive random node removal [43]. For each network, *η* values were calculated across sequential removal steps and normalized to the initial value (*η* = 1.0 at 0% removal) to allow comparison among vegetation types. A vulnerability index (V) was then computed as the complement of the area under the normalized connectivity curve, where higher V values indicate faster loss of connectivity and lower structural robustness [44]. This integrated analytical framework provided a quantitative basis for assessing community assembly mechanisms and evaluating the stability and resilience of microbial co-occurrence networks along the forest–wetland ecotone.

### 2.7. Functional Prediction

Functional potentials of soil microbial communities were predicted using PICRUSt2 (version 2.5.0, University of Connecticut, Storrs, CT, USA) based on ASV tables generated by DADA2 (version 1.28, University of California, San Francisco, CA, USA). ASVs with relative abundances below 0.01% were removed prior to prediction to minimize noise. The prediction accuracy was assessed using the Nearest Sequenced Taxon Index (NSTI), and ASVs with NSTI values greater than 2.0 were excluded from downstream analyses to ensure reliability. Predicted functional profiles were assigned to KEGG Orthologs (KOs) and summarized at both KEGG Level 2 categories and individual pathway levels, with special emphasis on nitrogen metabolism. Relative abundances were normalized prior to comparison to control for differences in sequencing depth. Statistical differences among vegetation types were tested using one-way ANOVA followed by Tukey’s HSD post hoc test in R software, with *p*-values adjusted for multiple testing using the false discovery rate (FDR) correction. To improve interpretability, predicted functions were cross-validated with qPCR results of key nitrogen-cycling genes. In addition, functional annotations were complemented using FAPROTAX to infer ecological roles and BugBase to assess microbial phenotypic traits, including anaerobic capacity and stress tolerance. These combined approaches provided an integrative overview of the metabolic potential and ecological adaptations of microbial communities across the forest–wetland ecotone.

### 2.8. Statistical Analyses

All analyses were conducted in R version 4.3.2. Group differences in soil properties, enzyme activities, and functional gene abundances were tested using one-way ANOVA followed by Tukey’s HSD; when multiple comparisons were involved, *p*-values were adjusted by the false discovery rate (FDR). Statistical significance was indicated in figures by distinct lowercase letters (*p* < 0.05) or asterisks (* *p* < 0.05, ** *p* < 0.01, *** *p* < 0.001). Community dissimilarities were visualized by principal coordinate analysis (PCoA, Bray–Curtis), and relationships between community structure, soil variables, and enzyme activities were examined by redundancy analysis (RDA) using the vegan package. Beta diversity was partitioned into replacement (Repl) and richness difference (RichDif) components with the betapart package. Random forest models were used to rank environmental predictors of nitrogen-cycling gene distributions. To address spatial structure, sampling followed a fixed transect with replicated plots within each vegetation type to minimize within-type spatial autocorrelation. Given the categorical design and limited replicates, mixed-effects models were not applied; inference focused on among-type contrasts using ANOVA and multivariate ordinations. Community assembly mechanisms were inferred under a standard null-model framework: |βNTI| > 2 indicates deterministic selection; when |βNTI| ≤ 2, RC_bray_ > 0.95 indicates dispersal limitation, RC_bray_ < −0.95 indicates homogenizing dispersal, and |RC_bray_| ≤ 0.95 indicates drift or undominated processes [45]. Consistent with common practice, βNTI and RC_bray_ were interpreted as heuristic indicators of assembly tendencies rather than definitive mechanistic proof.

## 3. Results

### 3.1. Functional Gene Abundance and Multivariate Variation

**Figure 1:**
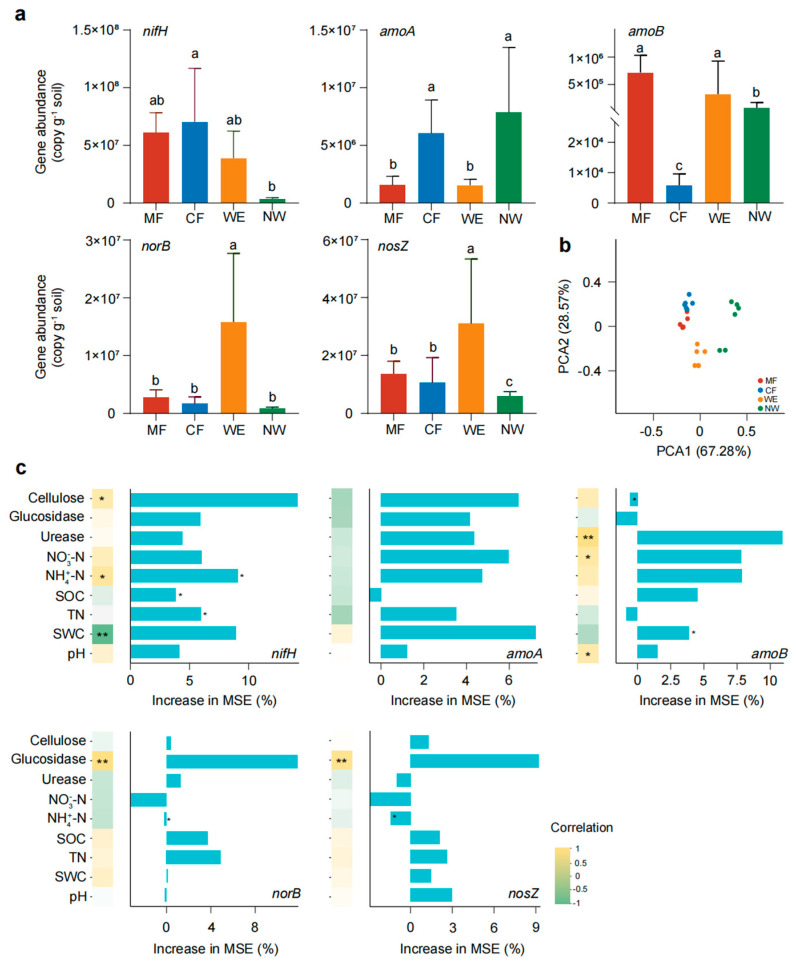
Abundance and drivers of nitrogen-cycling functional genes across four vegetation types. (**a**) Changes in the abundance of functional genes mediating different nitrogen-cycling processes. Different lowercase letters above bars indicate significant differences among vegetation types (*p* < 0.05). (**b**) Distribution of nitrogen-cycling gene abundance under different vegetation types. Each vegetation type includes six independent plots (*n* = 6). (**c**) Random forest and correlation analyses of nitrogen-cycling functional gene abundance with environmental variables. MSE represents the percentage increase in mean squared error, indicating the relative importance of each variable. Colors indicate the sign and magnitude of the correlation between soil properties and target genes. Asterisks within cells denote significance levels (* *p* < 0.05, ** *p* < 0.01).

As shown in Figure 1a, the abundances of nitrogen-cycling functional genes varied significantly among vegetation types along the forest–wetland ecotone. The *nifH* gene was significantly more abundant in CF than in MF, WE, and NW (*p* < 0.05). The *amoA* gene exhibited higher abundance in CF and NW than in MF and WE (*p* < 0.05), whereas *amoB* was significantly enriched in MF, WE, and NW relative to CF (*p* < 0.05). Both *norB* and *nosZ* showed the highest abundances in WE soils (*p* < 0.05). PCA (Figure 1b) revealed a clear clustering of samples by vegetation type, with PC1 and PC2 jointly explaining 95.85% of total variance. This pattern highlights the strong differentiation of nitrogen-cycling assemblages across vegetation transitions. Model-based importance analysis (Figure 1c) identified distinct environmental and biochemical drivers: the *nifH* gene showed a significant negative correlation with SWC (*p* < 0.01) and positive correlations with NH_4_^+^–N and cellulase activity (*p* < 0.05). In contrast, the *amoB* gene was positively correlated with urease activity (*p* < 0.01), NO_3_^−^–N, and pH (*p* < 0.05). The *norB* and *nosZ* genes were driven primarily by β-glucosidase (*p* < 0.01). These results indicate that hydrological and enzymatic controls, rather than a monotonic spatial gradient, govern the distribution of nitrogen-cycling genes across the four vegetation types.

### 3.2. Microbial Diversity and Community Composition Along the Vegetation Transitions

**Figure 2:**
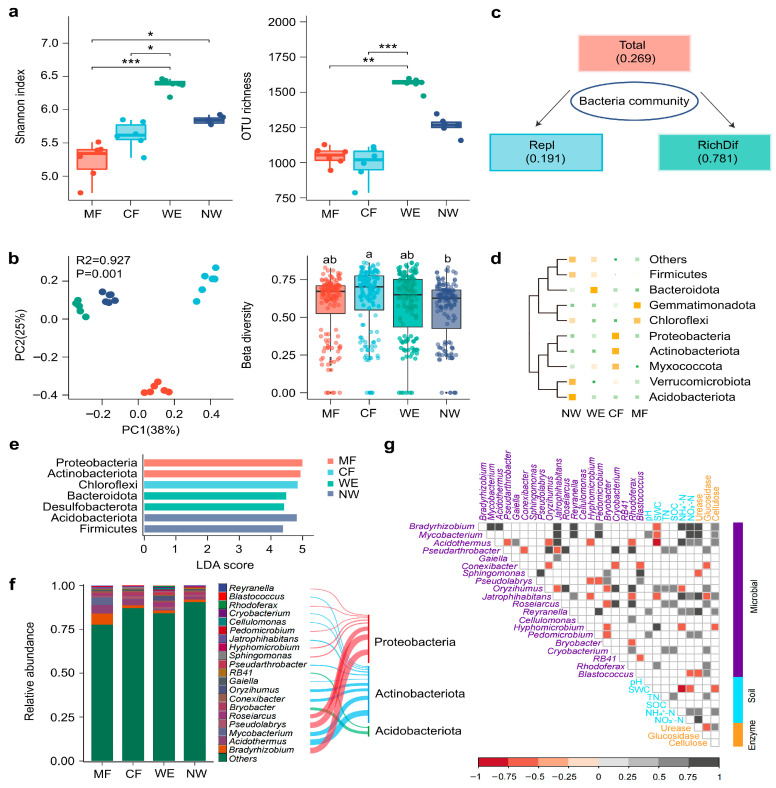
Microbial diversity and community composition along the forest–wetland transitions. Variation in bacterial (**a**) α-diversity and (**b**) β-diversity. (**c**) Partitioning of bacterial β-diversity (Repl, replacement component; RichDif, richness difference component). (**d**) Composition of the soil bacterial community at the phylum level, showing the top 10 most abundant phyla. The lines represent a clustering tree constructed based on the similarity of microbial community compositions among samples, reflecting the degree of similarity in community structure between samples. (**e**) Indicator taxa with significant differences identified by LEfSe analysis. (**f**) Relative abundance of the top 20 genera and their corresponding phyla. (**g**) Correlations between dominant bacterial genera and environmental variables. Different lowercase letters above bars indicate significant differences among vegetation types (*p* < 0.05). Asterisks indicate statistically significant differences among vegetation types (* *p* < 0.05, ** *p* < 0.01, *** *p* < 0.001).

Microbial α-diversity indices varied significantly across vegetation types (*p* < 0.05). Shannon diversity and OTU richness were significantly higher in WE compared with MF, CF, and NW (Figure 2a). PCoA based on Bray–Curtis distances revealed distinct clustering of microbial communities across vegetation transitions, with MF and CF positioned closer together, while WE and NW formed separate clusters. PC1 and PC2 together explained 63% of the total variance, highlighting compositional differentiation along the ecotone (Figure 2b). Beta diversity values were significantly lower in NW compared with CF (*p* < 0.001). For bacterial community assembly (Figure 2c), the contribution of richness differences (RichDif, 0.781) was much greater than that of replacement (Repl, 0.191), with a total dissimilarity value of 0.269. At the phylum level, hierarchical clustering of community composition (Figure 2d) grouped NW and WE together and CF and MF together, indicating clear compositional differences between wetland-associated and forest-associated communities. Myxococcota, Actinobacteriota, Proteobacteria, Chloroflexi, and Gemmatimonadota were primarily associated with forest soils (CF and MF), whereas Acidobacteriota, Verrucomicrobiota, Bacteroidota, and Firmicutes were representative of wetland soils (NW and WE). LEfSe identified vegetation-specific phylum-level biomarkers, with Proteobacteria and Bacteroidota discriminating MF, Chloroflexi discriminating CF, Bacteroidota together with Desulfobacterota discriminating WE, and Acidobacteriota plus Firmicutes discriminating NW, as supported by their higher LDA score (Figure 2e). At the genus level, MF and CF soils contained higher abundances of *Reyranella* (3.1%), *Blastococcus* (2.7%), and *Gaiella* (2.5%), whereas *Bryobacter* (2.9%), *Rhodoferax* (3.5%), and *Bradyrhizobium* (2.8%) were enriched in MF (Figure 2f). Heatmap analysis confirmed distinct distribution patterns across the vegetation transitions (Figure 2g).

### 3.3. Microbial Community Differentiation and Assembly Patterns Across the Vegetation Transitions

**Figure 3:**
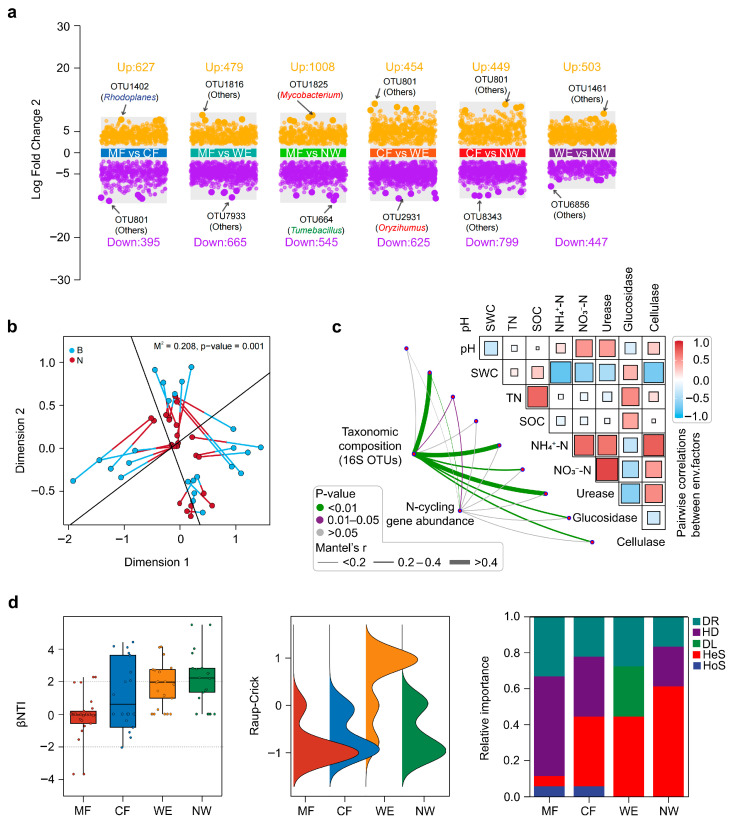
Microbial diversity and community composition along the forest–wetland transitions. (**a**) Differential abundance analysis showing significant OTU shifts among vegetation types. (**b**) Procrustes analysis showing the concordance between soil bacterial community structure (B) and N-cycling gene abundance (N) across vegetation types, with black lines separating MF, CF, WE, and NW, where the distance between paired points indicates the degree of compositional similarity. (**c**) Mantel test results showing correlations between microbial community composition, nitrogen-cycling functional genes, and environmental factors. (**d**) Community assembly processes based on βNTI and RCbray. Categories include heterogeneous selection, homogeneous selection, dispersal limitation, homogenizing dispersal, and drift/undominated, defined as: |βNTI| > 2 selection; |βNTI| ≤ 2 & RC_bray_ > +0.95 dispersal limitation; |βNTI| ≤ 2 & RC_bray_ < −0.95 homogenizing dispersal; otherwise drift/undominated, including homogeneous selection (HoS), heterogeneous selection (HeS), dispersal limitation (DL), homogenizing dispersal (HD), and drift (DR).

Differential abundance analysis (Figure 3a) revealed distinct enrichment patterns of key OTUs across vegetation types. *Rhodoplanes* was significantly enriched in MF relative to CF, whereas *Mycobacterium* showed higher abundance in MF than in NW. In contrast, *Geobacter* and *Anaerolinea* were differentially enriched in WE and NW, respectively, reflecting habitat-specific adaptation among dominant taxa. Pairwise comparisons identified 449–1008 significantly enriched and 395–799 depleted OTUs, underscoring pronounced community differentiation along the vegetation transitions. Procrustes analysis (Figure 3b) demonstrated a significant concordance between bacterial community composition (ordination based on 16S rRNA ASVs) and nitrogen cycling functional gene profiles (M^2^ = 0.208, *p* = 0.001). Mantel tests (Figure 3c) further confirmed strongest predictors for community composition, functional genes, and environmental drivers (*p* < 0.01), with SOC, TN, SWC, and urease activity identified as the strongest predictors. Random forest analysis (10,000 trees; cross-validation R^2^ = 0.79) ranked SOC, TN, and SWC as major predictors of *nifH* and *amoA*, whereas cellulase, β-glucosidase, and SOC were the strongest predictors for *norB* and *nosZ*, emphasizing the linkage between carbon turnover and nitrogen functional potential. Community assembly analysis using βNTI and RC_bray_ metrics (Figure 3d) revealed contrasting ecological processes among vegetation types. MF was dominated by stochastic processes (βNTI ≈ 0; low RC_bray_), CF exhibited a mixed influence of deterministic and stochastic factors, WE showed high RC_bray_ dissimilarity, indicating strong dispersal limitation, whereas NW exhibited the lowest level of stochastic influence.

### 3.4. Microbial Co-Occurrence Networks and Functional Associations Across Vegetation Types

**Figure 4:**
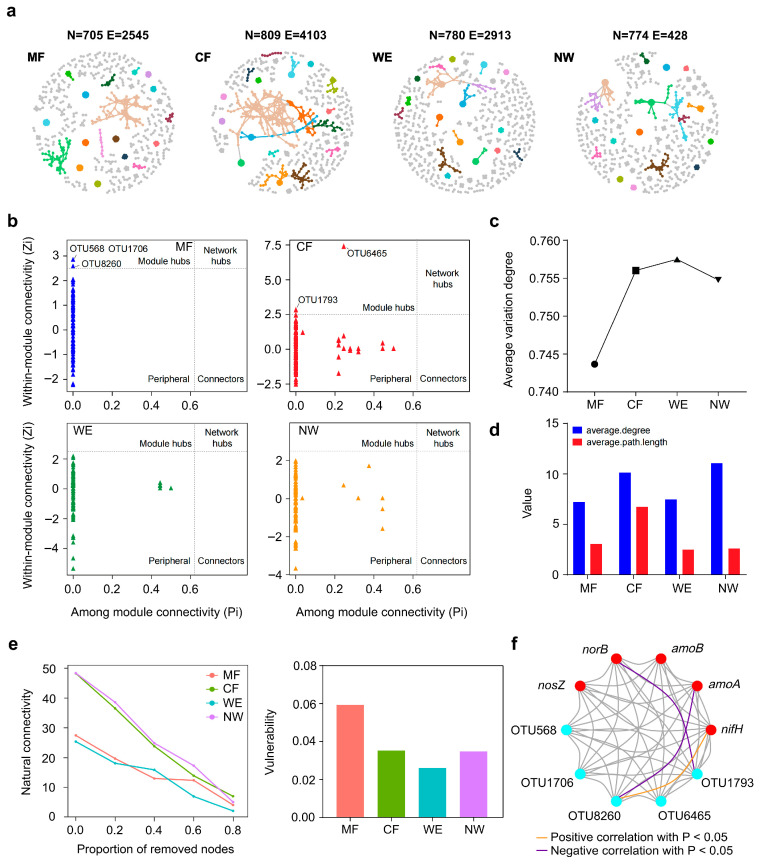
Microbial co-occurrence networks and functional associations across the forest–wetland transitions. (**a**) Co-occurrence network analysis of soil bacterial communities, with N and E representing the number of nodes and edges, respectively. Node colors represent modularity classes identified by the Louvain algorithm, where nodes of the same color belong to the same topological module; edge thickness denotes correlation strength. (**b**) Identification of key OTUs in network construction based on Zi–Pi analysis. (**c**) Community variability assessed by the Average Variation Degree (AVD) index. (**d**) Changes in network average degree and average path length among vegetation types. (**e**) Normalized natural connectivity (*η*_norm_) plotted against the fraction of nodes removed; the vulnerability index (V) is derived from the area under the *η*_norm_ curve, where larger values indicate lower robustness. (**f**) Correlations between keystone taxa in the co-occurrence network and N-cycling gene abundance.

The CF exhibited the largest and densest network, followed by WE and MF, whereas NW contained a comparable number of nodes to WE but substantially fewer edges. This pattern aligns with the α-diversity pattern (Figure 2a), where CF and WE also exhibited higher diversity, implying that enhanced taxonomic richness supports greater co-occurrence complexity. Highly modular networks typically display reduced global connectivity at baseline but greater within-module stability, consistent with ecological partitioning among vegetation types. In the Zi–Pi plots (Figure 4b), MF was dominated by peripheral nodes with three module hubs (OTU568, OTU1706, OTU8260). CF showed higher topological complexity, including one module hub (OTU1793) and numerous peripheral taxa. WE and NW were mainly composed of peripheral nodes, and no evident hubs or connectors were detected. The Average Variation Degree (Figure 4c) showed minor variation among vegetation types, being lowest in MF, increasing through CF, peaking in WE, and slightly decreasing in NW, reflecting moderate community variability along the transitions. Network-level metrics (Figure 4d) showed that CF and NW had higher average degrees than MF and WE, whereas the average path length was longest in CF, intermediate in MF, and shortest in WE and NW, suggesting vegetation-dependent variation in network compactness and connectivity efficiency. Although MF and WE exhibited lower initial natural connectivity than CF and NW (Figure 4e), this reflects their more modular and compartmentalized structures rather than a lower total number of edges. Network stability analysis further showed that natural connectivity declined progressively with node removal in all vegetation types, with CF and NW retaining higher connectivity under disturbance, whereas MF and WE declined more rapidly. Moreover, no significant hub-associated correlations were detected in WE or NW, suggesting that functional linkages between network topology and nitrogen-cycling genes were spatially restricted across vegetation transitions. Vulnerability analysis indicated that MF had the highest vulnerability, NW an intermediate level, while CF and WE maintained lower vulnerability and greater structural robustness, consistent with their slower connectivity decline. Finally, correlation analysis (Figure 4f) identified a single OTU in MF showing significant but opposite correlations with *nifH* and *amoA*, indicating differentiated coupling between nitrification-related taxa and functional potential.

### 3.5. Functional Predictions of Microbial Communities Along the Forest–Wetland Transitions

**Figure 5:**
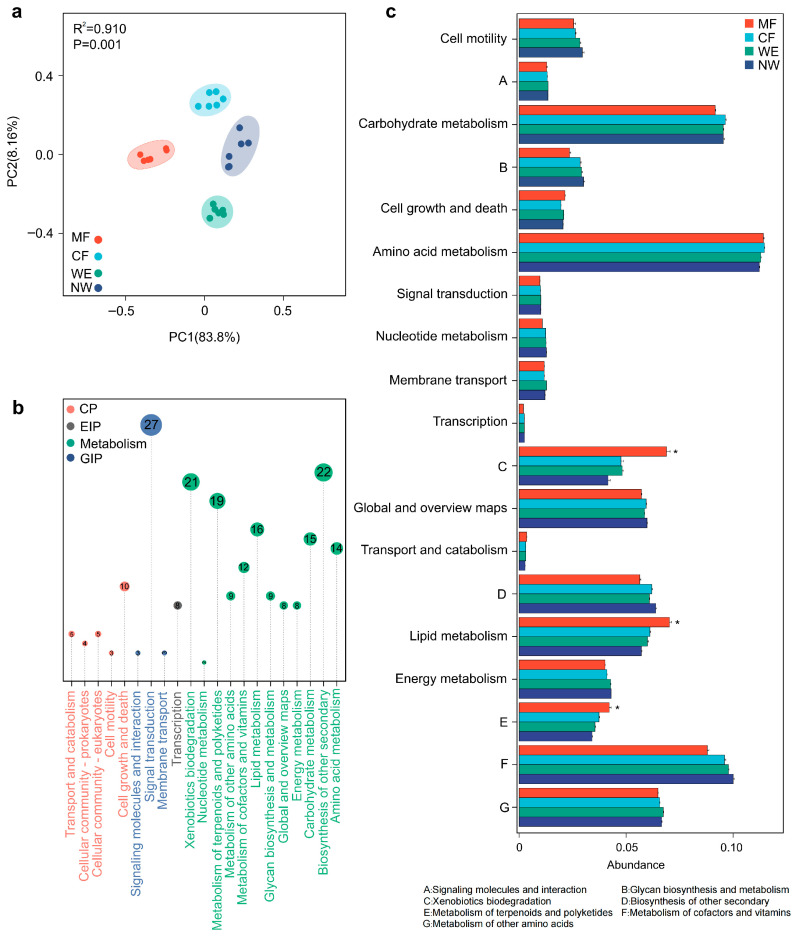
Predicted functional profiles of microbial communities across the forest–wetland transitions. (**a**) PCoA based on predicted functional profiles showing treatment effects. (**b**) KEGG Level 2 functional category distributions and the number of associated Level 3 pathways (CP: Cellular Processes; EIP: Environmental Information Processing; GIP: Genetic Information Processing). Note: Colors in panel (**b**) represent KEGG functional categories and are independent of the vegetation-type color coding used in other panels, to facilitate visual distinction of functional groupings. (**c**) Differential abundances of KEGG Level 2 pathways among treatments (* *p* < 0.05). Statistical significance was assessed at both KEGG Level 2 and pathway levels using one-way ANOVA followed by Tukey’s HSD post hoc test, with *p*-values adjusted by the false discovery rate (FDR); results with adjusted *p* < 0.05 were considered significant.

Predicted functional profiles revealed distinct metabolic adaptations of microbial communities along the vegetation transitions. PCoA analysis (Figure 5a) demonstrated clear separation among vegetation types, indicating strong divergence in metabolic potential. The distribution of KEGG functional categories (Figure 5b) showed that metabolism contained the largest number of pathways (153), followed by environmental information processing (33), cellular processes (27), and genetic information processing (8). Within metabolism, subcategories related to xenobiotic biodegradation (21), lipid metabolism (16), amino acid metabolism (14), and energy metabolism (8) were most abundant. Comparisons of secondary functional categories (Figure 5c) further revealed that MF soils exhibited significantly higher relative abundances of xenobiotic biodegradation, lipid metabolism, and terpenoid/polyketide metabolism than CF, WE, and NW (*p* < 0.05).

## 4. Discussion

The transition from forest to wetland across the ecotone is accompanied by a fundamental reorganization of soil microbial communities and their nitrogen cycling functions. As soils shift from well-drained, aerated forest conditions to waterlogged wetlands, the dominant N-transforming processes change accordingly. In forest soils, the microbial community shows higher abundances of genes for *nifH* and *amoA*, indicating active N inputs and conversions under oxygen-rich, organic matter-rich conditions [46]. By contrast, saturated wetland soils are enriched in denitrification genes (particularly *nosZ*), reflecting microbial communities geared towards complete denitrification under anaerobic conditions [47]. These functional shifts are driven by the stark differences in soil resources and redox conditions along the gradient: forests with higher organic carbon and nitrogen availability promote diverse diazotrophs and nitrifiers, whereas waterlogged wetlands with lower redox potential favor specialized anaerobic denitrifiers [48,49]. Ecologically, this leads to different fates of nitrogen and greenhouse gases in the two habitats [50]. Wetland soils, with their abundance of *nosZ* carrying microbes, have greater potential to reduce nitrous oxide (N_2_O) to inert N_2_, thereby mitigating N_2_O emissions and preventing nitrate accumulation through continual denitrification [51]. In contrast, forest soils dominated by nitrification can accumulate nitrate and may produce pulses of N_2_O when transient wet conditions occur, due to a more limited capacity for N_2_O reduction [52]. Thus, vegetation-driven changes in soil moisture and carbon availability tip the balance among N-fixation, nitrification, and denitrification across the forest–wetland ecotone, with broad implications for soil fertility and atmospheric N_2_O fluxes [53,54].

Vegetation transitions also lead to major shifts in microbial community diversity and composition along the ecotone. Forest soils support higher microbial α-diversity and a more heterogeneous community structure than wetlands, owing to the varied litter inputs and stable aerobic niches in forests [55]. In the water-saturated wetlands, strong environmental filtering occurs under anoxic, nutrient-rich conditions, which lowers overall diversity and selects for a few specialized anaerobic taxa adapted to flooded soils [56]. In other words, the gradient in oxygen and moisture availability acts as a sieve: well-aerated forest habitats allow many microbial groups to coexist, whereas waterlogged wetland habitats restrict community membership to those that can tolerate or thrive in anaerobic conditions [57]. Accordingly, the dominant taxa differ markedly between the two ends of the ecotone. Forest communities are enriched in bacterial groups capable of aerobic decomposition (e.g., Actinobacteriota and certain Proteobacteria), taking advantage of high organic matter inputs under oxic conditions [58]. In contrast, wetland communities are dominated by taxa such as Bacteroidota and anaerobic *Deltaproteobacteria* (e.g., *Geobacter*), which are well suited for anaerobic respiration and fermentation in waterlogged soils [59]. These taxonomic shifts align with differences in soil chemistry: the higher soil organic C and nutrient levels in forests sustain a diverse cohort of aerobic heterotrophs, whereas the anoxic, carbon-rich wetland soils favor microbes that can utilize alternative electron acceptors and fermentative pathways [60]. This reassembly of the microbiome has significant functional consequences. The greater taxonomic and functional diversity in forest soil communities likely confers higher ecosystem multifunctionality and resilience, as multiple taxa can redundantly perform key processes [61]. Meanwhile, the lower diversity but highly specialized composition of wetland communities means they efficiently carry out certain processes (such as complete denitrification) but with less functional redundancy [62]. The forest–wetland ecotone thus emerges as a dynamic hotspot of microbial turnover: even small shifts in hydrology or substrate availability can cause disproportionate changes in community structure, making this transition zone both vulnerable to disturbances and crucial for regulating nutrient cycling and greenhouse gas fluxes in the broader landscape [63,64].

In tandem with changes in community composition, vegetation-induced habitat differences alter the prevailing mechanisms of microbial community assembly along the gradient [65]. The strong niche contrast between the forest (upland) and wetland (waterlogged) environments results in distinct sets of taxa thriving in each, reflecting different balances of deterministic versus stochastic assembly processes [66]. In the relatively stable, resource-rich forest soils, deterministic processes (niche-based selection) dominate community assembly organisms best adapted to the high-organic, oxic conditions competitively exclude others, yielding more predictable and structured communities [67]. By contrast, the saturated and fluctuating conditions of wetlands increase the influence of stochastic factors such as chance colonization, ecological drift, and dispersal limitation in shaping the community [68]. This shift in assembly regime is evidenced by our observations that key soil properties modified by vegetation (e.g., soil organic C, total N, pH, moisture) were strongly correlated with microbial community composition and functional gene patterns, indicating a significant role of environmental filtering; yet, null model analyses revealed higher stochasticity in wetland communities compared to forests [69,70]. In practical terms, forest soil microbiomes are more consistently structured by specific environmental conditions, whereas wetland microbiomes exhibit greater random temporal variability and compositional turnover driven by unpredictable events [71]. Consequently, forest ecosystems tend to maintain a more stable and tightly regulated microbiome, while wetland soil communities may be more sensitive to disturbance and subject to greater fluctuations over time [72]. Both deterministic and stochastic processes are at play in each system, but their relative contributions shift with vegetation and hydrology [73]. This highlights how vegetation transitions modulate community assembly mechanisms with consequences for ecosystem stability and function.

Vegetation change not only influences which microbes are present, but also how they interact and what functional capabilities they collectively harbor [74]. In forest soils, the microbial community formed a much more complex and interconnected co-occurrence network, whereas in wetlands the network was comparatively sparse and fragmented [75]. The high diversity and heterogeneous resources in forests foster dense microbial networks with many positive co-occurrences and multiple keystone taxa acting as hubs, which likely confers stability through functional redundancy [76]. By contrast, the anoxic and stressful wetland conditions lead to simplified networks with far fewer connections and only a few keystone species, indicating a community with lower redundancy and more isolated clusters of specialists [77]. This decline in network complexity and connectivity from forest to wetland suggests that wetland microbial communities are more fragile and prone to disruption if key taxa are lost [78]. Notably, the identity of keystone taxa in each habitat mirrored the dominant nutrient cycling processes. In forest soil networks, the hub taxa were often associated with nitrification and N-fixation functions, implicating them in sustaining soil nitrogen availability [79]. In wetland networks, the keystone microbes were linked to denitrification genes, reflecting their role in mediating greenhouse gas production and nitrogen removal [80]. These findings demonstrate that vegetation transitions reorganize not only microbial composition but also microbe–microbe interactions and functional linkages [81]. Consistently, the metabolic potential of the soil microbiome shifts with the change in vegetation and hydrology. Forest soils are enriched in metabolic pathways related to biosynthesis and aerobic energy metabolism (e.g., pathways for carbohydrate and amino acid metabolism), consistent with vigorous organic matter decomposition under oxic conditions [82]. In contrast, wetland communities show greater potential for anaerobic and stress-tolerant metabolisms, including pathways for fermentation, reductive respiration, and xenobiotic degradation, reflecting adaptation to prolonged waterlogging and limited oxygen [83]. In essence, as the habitat transitions from forest to wetland, microbial life strategies pivot from fast, oxygen-driven cycles to slower, anaerobic cycles optimized for saturated environments [84]. These functional differences have important implications at the ecosystem level. Wetland microbiomes, with enhanced denitrification and fermentation capacities, can remove more reactive nitrogen (mitigating nitrate leaching and N_2_O emissions) but also tend to channel carbon into more reduced end-products such as CH_4_ [85,86]. Meanwhile, forest soil communities, retaining a broader metabolic repertoire, likely support a wider range of ecosystem services from rapid nutrient recycling to soil organic matter accumulation thereby promoting higher multifunctionality and resilience in the face of environmental change [87]. The shift from a highly connected, functionally redundant microbial network in forests to a simpler, specialized network in wetlands underscores a trade-off between stability and efficiency that is orchestrated by hydrological conditions and nutrient regimes [88].

In summary, this study demonstrates that vegetation transitions across the forest–wetland ecotone profoundly reshape soil microbial diversity, community assembly processes, interaction networks, and functional potential. Forest soils exhibited higher microbial diversity, a greater prevalence of nitrogen-fixation and nitrification genes, and more complex co-occurrence networks, whereas wetland soils showed lower diversity, enrichment of denitrification-related genes, and simplified, less robust networks [89]. These differences were strongly associated with variation in soil moisture, redox status, and nutrient availability, linking vegetation-driven hydrological changes to shifts in microbial community structure and function [90]. Collectively, our findings highlight the ecological significance of forest–wetland ecotones as zones where sharp environmental gradients regulate nutrient cycling and greenhouse gas dynamics [91]. Such ecotonal areas can act as critical buffers for nutrient retention and N_2_O mitigation at the landscape scale, but they are also sensitive to disturbances and climate-induced hydrological shifts [92]. From an application perspective, understanding these microbial patterns provides valuable insights for ecosystem management. For instance, the promotion of plant communities that maintain higher soil aeration in marginal wetland areas could enhance microbial diversity and functional redundancy, improving ecosystem resilience [93]. Conversely, conserving natural wetland features is crucial for sustaining specialized microbes that perform complete denitrification, thereby reducing net N_2_O emissions [94]. The knowledge gained here can inform ecological restoration and sustainable land-use planning in black-soil ecotones, ensuring that both forest and wetland microbial functions are preserved to support soil health and climate regulation [95]. It should be noted that our functional predictions (based on 16S rRNA gene inference) reflect potential capacities rather than direct measurements of process rates. Future research should therefore integrate multi-omic approaches (metagenomics, metatranscriptomics) with in situ assays of nutrient transformations and greenhouse gas fluxes to verify and expand upon these findings [96]. Long-term monitoring of forest–wetland transition zones under changing hydrological regimes will also be essential for predicting the stability of these microbial processes as climate change alters the balance between upland and wetland ecosystems [97,98].

## 5. Conclusions

Vegetation transitions across the forest–wetland ecotone in Northeast China fundamentally reshape soil microbial diversity, assembly processes, interaction networks, and nitrogen-cycling functions. Rather than forming a smooth gradient, these transitions generate discrete hydrological and redox domains that act as ecological filters, driving microbial niche partitioning and functional differentiation. Forest soils, characterized by stable oxic and carbon-rich conditions, support highly diverse and interconnected microbial communities with strong potentials for nitrogen fixation and nitrification. In contrast, wetland soils select for specialized denitrification related taxa adapted to anoxic and electron-acceptor-limited environments. The wetland edge represents a dynamic ecological interface where alternating moisture and redox conditions foster the coexistence of nitrifiers and denitrifiers, thereby enhancing carbon–nitrogen coupling and sustaining functional redundancy. Network and functional analyses together reveal that microbial stability and nitrogen transformation capacity are primarily regulated by hydrological and biogeochemical heterogeneity, rather than by vegetation type alone. This emphasizes that the forest–wetland transition zone functions as a critical hotspot of nutrient cycling and greenhouse gas regulation. Understanding these mechanisms provides a scientific basis for predicting biogeochemical feedback and for developing targeted strategies to manage and restore black-soil ecotones under changing hydrological regimes.

## Data Availability

Data will be provided as requested.

## Supplementary Materials

The following supporting information can be downloaded at: https://www.mdpi.com/article/10.3390/biology14111474/s1, Figure S1: Map showing different vegetation location of the study. Table S1: The physicochemical properties of soil in different forest–wetland ecotones. Table S2: OTU abundance table of soil microbial communities across vegetation types.

## Abbreviations

The following abbreviations are used in this manuscript:

AK	Available Potassium
*amoA*	Ammonia Monooxygenase Subunit A Gene
*amoB*	Ammonia Monooxygenase Subunit B Gene
AP	Available Phosphorus
ASV	Amplicon Sequence Variant
βNTI	Beta Nearest Taxon Index
BG	β-glucosidase
CEL	Cellulase
CF	Coniferous Forest
EC	Electrical Conductivity
LDA	Linear Discriminant Analysis
LEfSe	Linear Discriminant Analysis Effect Size
MF	Mixed Forest
NH_4_^+^-N	Ammonium Nitrogen
NO_3_^−^-N	Nitrate Nitrogen
*norB*	Nitric Oxide Reductase Subunit B Gene
*nosZ*	Nitrous Oxide Reductase Gene
NW	Natural Wetland
OTU	Operational Taxonomic Unit
PCoA	Principal Coordinate Analysis
pH	Potential of Hydrogen
QIIME2	Quantitative Insights Into Microbial Ecology, version 2
RDA	Redundancy Analysis
SOC	Soil Organic Carbon
SWC	Soil Water Content
TN	Total Nitrogen
URE	Urease
WE	Wetland Edge
*nifH*	Nitrogen Fixation Gene
