# Supplementary material for: Ecotone-Driven Vegetation Transitions Reshape Soil Nitrogen Cycling Functional Genes in Black Soils of Northeast China
Source: Biology (Basel). 2025 Oct 23;14(11):1474. doi: 10.3390/biology14111474 (PMC12649909; doi:10.3390/biology14111474)
Supplement: Supplementary file 1 [file biology-14-01474-s001.zip › biology-3884175-supplementary/Supplementary Files/Supplementary Table S1.pdf]

Table S1. The physicochemical properties of soil in different forest–wetland ecotones.

Variables	MF	CF	WE	NW
pH	6.72 ± 0.36 a	6.41 ± 0.11 a	6.42 ± 0.08 a	6.33 ± 0.30 b
SWC (%)	30.17 ± 1.85 b	32.79 ± 1.85 b	47.57 ± 2.77 a	48.01 ± 2.95 a
TN ($g \cdot kg^{-1}$)	3.54 ± 0.21 b	3.31 ± 0.34 b	4.88 ± 1.42 a	2.54 ± 0.85 b
SOC ($g \cdot kg^{-1}$)	30.12 ± 5.93 ab	25.52 ± 6.40 b	39.39 ± 6.64 a	25.32 ± 7.80 b
NH_4^+-N ($mg \cdot kg^{-1}$)	48.49 ± 7.41 a	43.92 ± 2.80 a	24.08 ± 1.47 b	24.92 ± 7.80 b
NO_3^--N ($mg \cdot kg^{-1}$)	24.79 ± 4.11 a	13.68 ± 2.77 b	11.99 ± 1.50 b	14.46 ± 2.23 b
Urease ($mg \cdot g^{-1} \cdot d^{-1}$)	11840.17 ± 963.47 a	5589.01 ± 652.96 c	5881.83 ± 499.07 c	7663.67 ± 498.84 b
β-Glucosidase ($mg \cdot g^{-1} \cdot d^{-1}$)	26542.50 ± 5029.70 b	30732.83 ± 2794.36 b	39304.00 ± 3320.78 a	26515.36 ± 1598.66 b
Cellulase ($\mu g \cdot g^{-1} \cdot d^{-1}$)	19.45 ± 1.18 a	19.60 ± 3.83 b	13.08 ± 2.44 b	5.08 ± 1.23 c

Note: values represent mean ± standard deviations (n = 6). Different letters indicate significant differences at $P < 0.05$ based on Tukey's HSD test. Abbreviations of soil properties: pH, soil pH value; SOC, soil organic carbon; TN, total nitrogen; SWC, soil water content; NH_4^+-N, ammonium nitrogen; NO_3^--N, nitrate nitrogen; AP, available phosphorus; AK, available potassium. MF, mixed forest; CF, coniferous forest; WE, wetland edge; NW, natural wetland.
