# Supplementary figures and images for: Ecotone-Driven Vegetation Transitions Reshape Soil Nitrogen Cycling Functional Genes in Black Soils of Northeast China
Source: Biology (Basel). 2025 Oct 23;14(11):1474. doi: 10.3390/biology14111474 (PMC12649909; doi:10.3390/biology14111474)

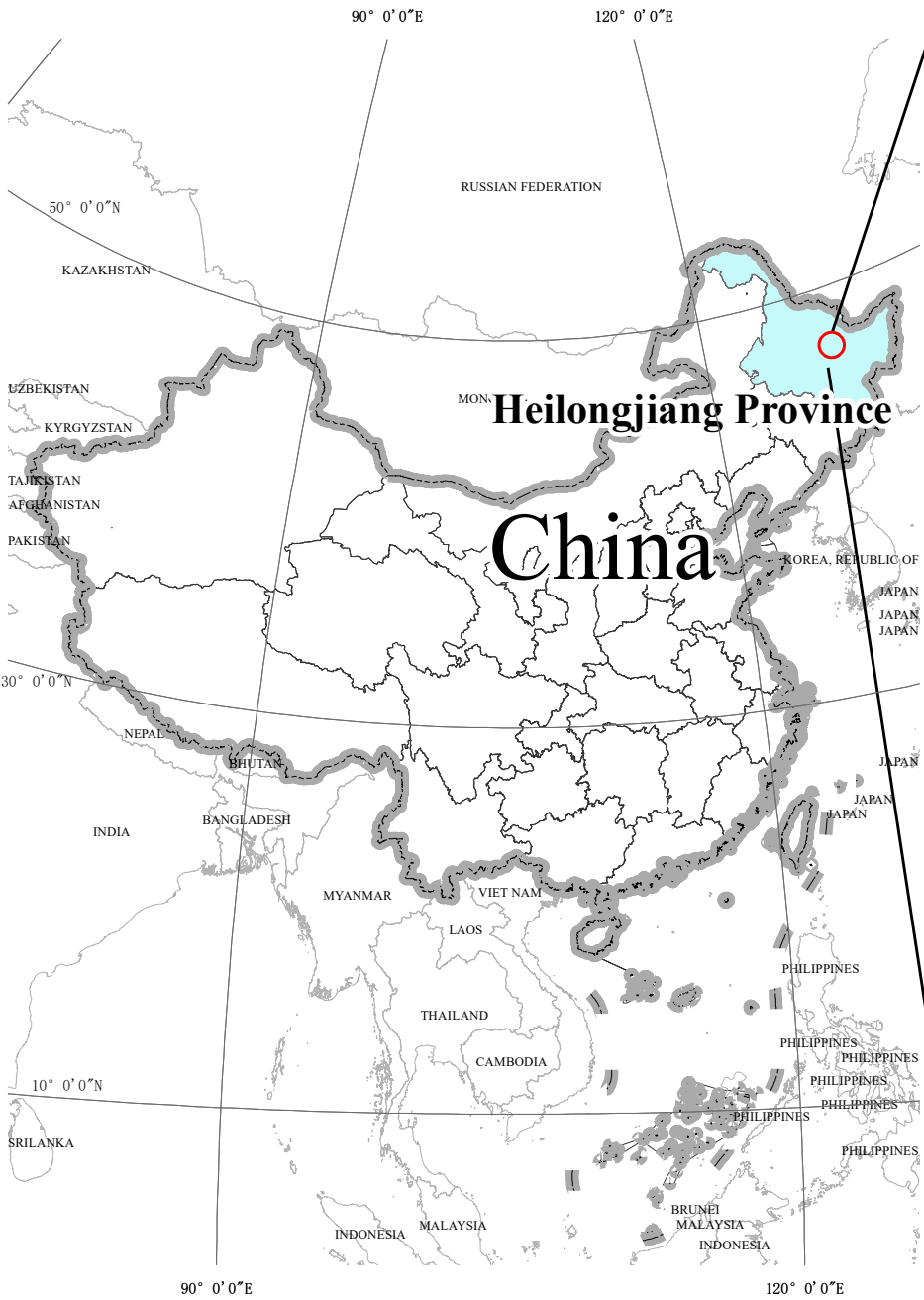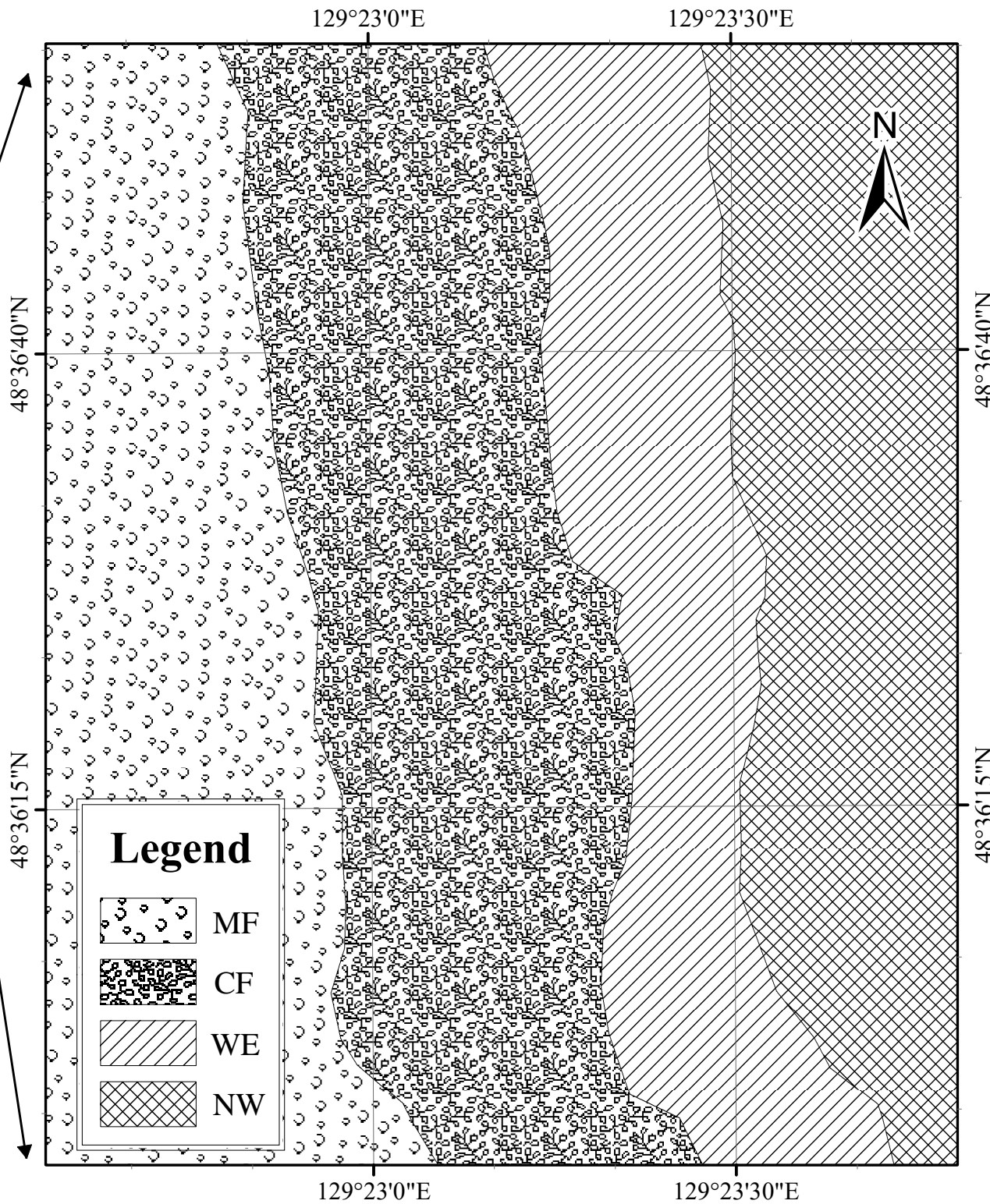

Supplement: Supplementary file 1 [file biology-14-01474-s001.zip › biology-3884175-supplementary/Supplementary Files/Figure. S1.pdf]
